# Long-term outcome in patients with aneurysmal subarachnoid hemorrhage requiring mechanical ventilation

**DOI:** 10.1371/journal.pone.0247942

**Published:** 2021-03-12

**Authors:** Kevin Chalard, Vivien Szabo, Frederique Pavillard, Flora Djanikian, Cyril Dargazanli, Nicolas Molinari, Federico Manna, Vincent Costalat, Gerald Chanques, Pierre-Francois Perrigault

**Affiliations:** 1 Department of Critical Care Medicine and Anesthesiology (DAR C), Gui de Chauliac University Hospital of Montpellier, Montpellier, France; 2 IGF, Univ. Montpellier, CNRS, Inserm, Montpellier, France; 3 Department of Neuroradiology, Gui de Chauliac University Hospital of Montpellier, Montpellier, France; 4 IMAG, CNRS, Univ Montpellier, CHU Montpellier, Montpellier, France; 5 Epidemiology and Clinical Research Department, University Hospital of Montpellier, Montpellier, France; 6 Department of Critical Care Medicine and Anesthesiology (DAR B), Saint Eloi University Hospital of Montpellier and PhyMedExp, University of Montpellier, INSERM, CNRS, Montpellier, France; Wayne State University, UNITED STATES

## Abstract

**Background:**

Patients affected with aneurysmal subarachnoid hemorrhage (aSAH) often require intensive care, and then present distinctive outcome from less severe patients. We aimed to specify their long-term outcome and to identify factors associated with poor outcome.

**Methods:**

We conducted a retrospective study in a French university hospital intensive care unit. Patients with aSAH requiring mechanical ventilation hospitalized between 2010 and 2015 were included. At least one year after initial bleeding, survival and degree of disability were assessed using the modified Rankin Scale (mRS) via telephone interviews. A multivariable logistic regression analysis was performed to determine independent factors associated with poor outcome defined as mRS≥3.

**Results:**

Two-hundred thirty-six patients were included. Among them, 7 were lost to follow-up, and 229 were analyzed: 73 patients (32%) had a good outcome (mRS<3), and 156 (68%) had a poor outcome (mRS≥3). The estimated 1-year survival rate was 63%. One-hundred sixty-three patients patients (71%) suffered from early brain injuries (EBI), 33 (14%) from rebleeding, 80 (35%) from vasospasm and 63 (27%) from delayed cerebral ischemia (DCI). Multivariable logistic regression identified independent factors associated with poor outcome including delay between aSAH diagnosis and mRS assessment (OR, 0.96; 95% CI, 0.95-0.98; p<.0001), age (OR per 10 points, 1.57; 95% CI, 1.12-2.19; p = 0.008), WFNS V versus WFNS III (OR, 5.71; 95% CI 1.51-21.61; p = 0.004), subarachnoid rebleeding (OR, 6.47; 95% CI 1.16-36.06; p = 0.033), EBI (OR, 4.52; 95% CI 1.81-11.29; p = 0.001) and DCI (OR, 4.73; 95% CI, 1.66-13.49; p = 0.004).

**Conclusion:**

Among aSAH patients requiring assisted ventilation, two-third of them survived at one year, and one-third showed good long-term outcome. As it appears as an independant factor associated with poor outcome, DCI shoud retain particular attention in future studies beyond angiographic vasospasm.

## Introduction

Aneurysmal subarachnoid hemorrhage (aSAH) presented a stable incidence of 9-10/100 000 persons per year over the last decades [1, 2]. The young mean age of affliction of 52-55 years makes this stroke a devastating disease. Although decreasing during the last 40 years, death rate remains as high as 30-50% [1–5]. Survivors are frequently affected with long-term disability, one third of them needing life-long care, and their majority do not recover their previous health status, suffering from cognitive and/or behavioral impairments [1, 6–8]. The main determinant of poor outcome, usually defined as death or major disability, is the initial severity of the hemorrhage, estimated from clinical and radiological scores such as the one developed by the World Federation of Neurosurgeons (WFNS) [9–12].

Because of their critical condition, patients diagnosed with poor-grade aSAH (grade IV and V of the WFNS classification) are admitted in intensive care units (ICU) so that early brain injury (EBI), delayed cerebral ischemia (DCI), and other medical complications can be managed. EBI, a direct consequence of blood irruption in the subarachnoid space and the resulting raise in intracranial pressure (ICP), is indeed more frequent in this category of patients, and is strongly associated with poor outcome [13, 14].

Management of EBI usually includes sedation, head bed elevation, normocapniae maintenance, extraventricular derivation when hydrocephalus is associated, and can further consist of hypothermia and decompressive craniectomy when ICP remains uncontrolled [15, 16]. In such situations, mechanical ventilation is required. DCI, resulting in secondary neurological deficiencies, is a frequent complication of aSAH diagnosed in 30% of patients [2, 16–18]. Its incidence increases with WFNS score [16], and is associated with unfavorable outcome [2, 12, 16, 18]. Therapeutic options are few, and nimodipine still is the only drug improving prognosis. However, early management of DCI can reverse it, such that continuous monitoring for early diagnosis has become a standard of care.

Long term outcome in aSAH patients have mainly been explored in randomized control trials cohorts. Fewer data is available from the specific population of poor grade aSAH patients.

The MASH cohort gathers information on Aneurysmal Subarachnoid Hemorrhage patients hospitalized in the French university hospital of Montpellier. In this cohort we investigated long-term outcome and risk factors for poor outcome in critically ill patients admitted in ICU.

## Materials and methods

An analytic retrospective study was conducted in an academic center in Montpellier, France. This observational study was performed according to the STROBE guidelines.

### Study population

Adults patients with aSAH hospitalized in the neuro-ICU between January 2010 and December 2015 were recruited. Only patients requiring mechanical ventilation were included. All patients underwent a computed tomography angiography (CTA) at admission to confirm subarachnoid hemorrhage caused by aneurysm rupture. Patients with iatrogenic aneurysm rupture and patients lost to follow-up were excluded from the analysis.

### Definitions of neurological events

- **Early Brain Injuries**: early brain injuries (EBI) was defined as the presence, on a CT scan performed within 48-72 hours after the initial bleeding, of hypodensities or intraparenchymal hemorrhage caused either by initial aneurysm bleeding, ICP elevation, aneurysm-securing procedure or resulting from external ventricular drain (EVD) placement [13, 14, 19]. Brain-dead patients before day 5 were also considered as early brain injured patients.
- **Rebleeding**: rebleeding of aneurysm was defined as an increase in the amount of subarachnoid blood between the initial and secondarily performed CT scans.
- **Angiographic vasospasm**: transcranial Doppler was not used to diagnose vasospasm. Angiographic vasospasm was defined as a reduction in arteries diameter of at least one-third, measured on either CTA or digital subtraction angiography (DSA) [17, 20].
- **Delayed cerebral ischemia**: delayed derebral ischemia (DCI) was defined as a delayed clinical deterioration due to ischemia (focal neurological impairment or a decrease of at least 2 points on the GCS that lasts for at least 1 hour that cannot be attributed to another cause), or as a delayed cerebral infraction (infarction on CT scan or MRI scans performed within 6 weeks after SAH, absent from the scan performed between 24 and 48 hours after aneurysm occlusion, and not attributable to another cause: aneurysm-securing procedure or extraventricular derivation placement) [17].

### Management protocol

- **Conventional management**: Aneurysm exclusion was performed as soon as possible after hospital admission. Type of aneurysm securing procedure (coiling or clipping) was collegially decided by neurosurgeons and neuroradiologists. In case of hydrocephalus, an external ventricular drain was placed. Surgical procedures including intracerebral hemorrhage (ICH) evacuation or decompressive craniectomy were performed when necessary. All patients were treated according to international guidelines [21].
  A CT scan was systematically performed between 24 and 48 hours after aneurysm exclusion. Patients were sedated and assisted with mechanical ventilation because of their critical condition (coma with Glasgow Coma Scale under 9) or in order to prevent secondary brain damages by controlling oxygenation and ventilation (PaO2 ≥ 75mmHg, PaCO2 between 35 and 40mmHg) and to maintain body temperature between 36°C and 38°C. Midazolam (0.2 to 0.4mg/kg/h) was associated with sufentanil (0.2 to 0.4*μ*g/kg/h). In uncontrolled ICP elevation situation, we added continuous infusion of sodium thiopental (1.5 to 2.5mg/kg/h).
- **Management of vasospasm and delayed cerebral ischemia**: All patients received nimodipine, administered either orally every 4 hours in a total daily dose of 360mg, or intravenously in a dose of 2mg/h when the oral route was not available. Patients were under continuous nurse supervision, physical examination was performed at least twice a day by physicians, notably to detect clinical deterioration associated with DCI. Transcranial Doppler monitoring through the acoustic transtemporal window was performed at least twice a day to detect ultrasonographic vasospasm [22, 23]. Physicians suspected vasospasm when the mean flow velocity in the middle cerebral artery increased by ≥ 50cm/s. When delayed cerebral ischemia or ultrasonographic vasospasm was suspected, CT or MRI were used to diagnose angiographic vasospasm and/or delayed cerebral infraction [20].
  In patients with angiographic vasospasm and/or DCI, cerebral perfusion pressure was optimized with normovolemia and induction of hypertension when necessary. Normovolemia was obtained using static measures of left ventricular filling pressures with transthoracic echocardiography or, when necessary, using dynamic tests such as fluid-challenge focusing on blood pressure elevation and/or increase in the aortic systolic velocity-time integral. Hypertension was obtained using norepinephrine.
  Combined cerebral intra-arterial milrinone [24, 25] and nimodipine [26] infusions were administered as a rescue therapy in two conditions: 1) angiographic vasospasm associated with persistent clinical delayed cerebral ischemia and 2) persistent vasospasm in unconscious patients in whom a neurological exam was not possible. Total intra-arterial daily doses of 4 mg of milrinone and 3 mg of nimodipine were infused. In case of bilateral angiographic vasospasm, doses were halved for bilateral infusion. Treatment was administered every day until condition improvement, assessed with clinical, ultrasonographic, and scannographic parameters.
  As a last resort, percutaneous transluminal angioplasty (PTA) was performed when vasospasm concerned the proximal portion of the middle cerebral artery [27]. Most severe vasospasms (diffuse or recurrent) were treated with continuous intraveinous infusion of milrinone at 1mg/h [28]

### Patients characteristics

The following data were recorded for every patient: age, sex, tobacco use, Glasgow Coma Scale (GCS) on admission, Simplified Acute Physiology Score II (SAPSII), presence of intracerebral hemorrhage (ICH) and Fisher score. Type of aneurysm securing procedure and delay between aneurysm securing and initial bleeding were noted. Mechanical ventilation and ICU stay durations were recorded.

### Long-term outcome

Degree of disability/dependence and survival were measured using the modified Rankin Scale (mRS) [29]. All interviews were performed by telephone at least one year after the initial bleeding. The elapsed time (delay, in months) between SAH diagnosis and mRs assessment (i.e. death or telephone call) was noted. A simplified standardized survey adapted to telephone exchanges was used [30]. The same investigator (KC) made all phone calls during a 4-months period. When patients were unable to reply to the survey, a caregiver or the patient’s physician was interviewed. A long-term outcome score indicating worse than slight disability (mRS ≧ 3) was considered as poor neurological outcome.

### Ethics and consent

This retrospective research was approved by our local Ethics Committee (Comite de Protection des Personnes Sud-Mediterannee IV, Montpellier, France; ID: Q-2015-09-07). Since there was no invasive procedure added to French ICUs standard of care, only verbal consent was required from the patient or the relatives, according to French law [31]. Patients contacted by telephone gave their oral consent.

### Statistical analysis

All variables studied were primarily selected by their clinical meaning and their significance in the prior literature. Data are presented as numbers with percentages for categorical variables, as means ± standard deviations (SD) or as medians with interquartile ranges for continuous variables. The Shapiro-Wilks test was used to evaluate normal distribution of continuous variables. Differences in demographic and clinical factors were compared using a chi square test or Fisher’s exact test for categorical variables, Student’s t Test or Mann-Whitney U test for continuous variables. The uncertainty in differences were described with 95% confidence intervals (95% CI). Covariables defined as binary variables and continuous variables were tested in a multivariable logistic regression model. The variables were selected in a stepwise selection procedure if p-value<0.15 in the univariable analysis then presented as adjusted odds ratios with 95% CI. The Hosmer-Lemeshow test was done to evaluate the goodness of fit in the final logistic regression model. We estimated the survival rates using the Kaplan-Meier method. Statistical analysis was performed with SAS v9 (SAS Institute, Cary, NC, USA).

## Results

### Patients’ demographic and clinical data

**Fig 1:**
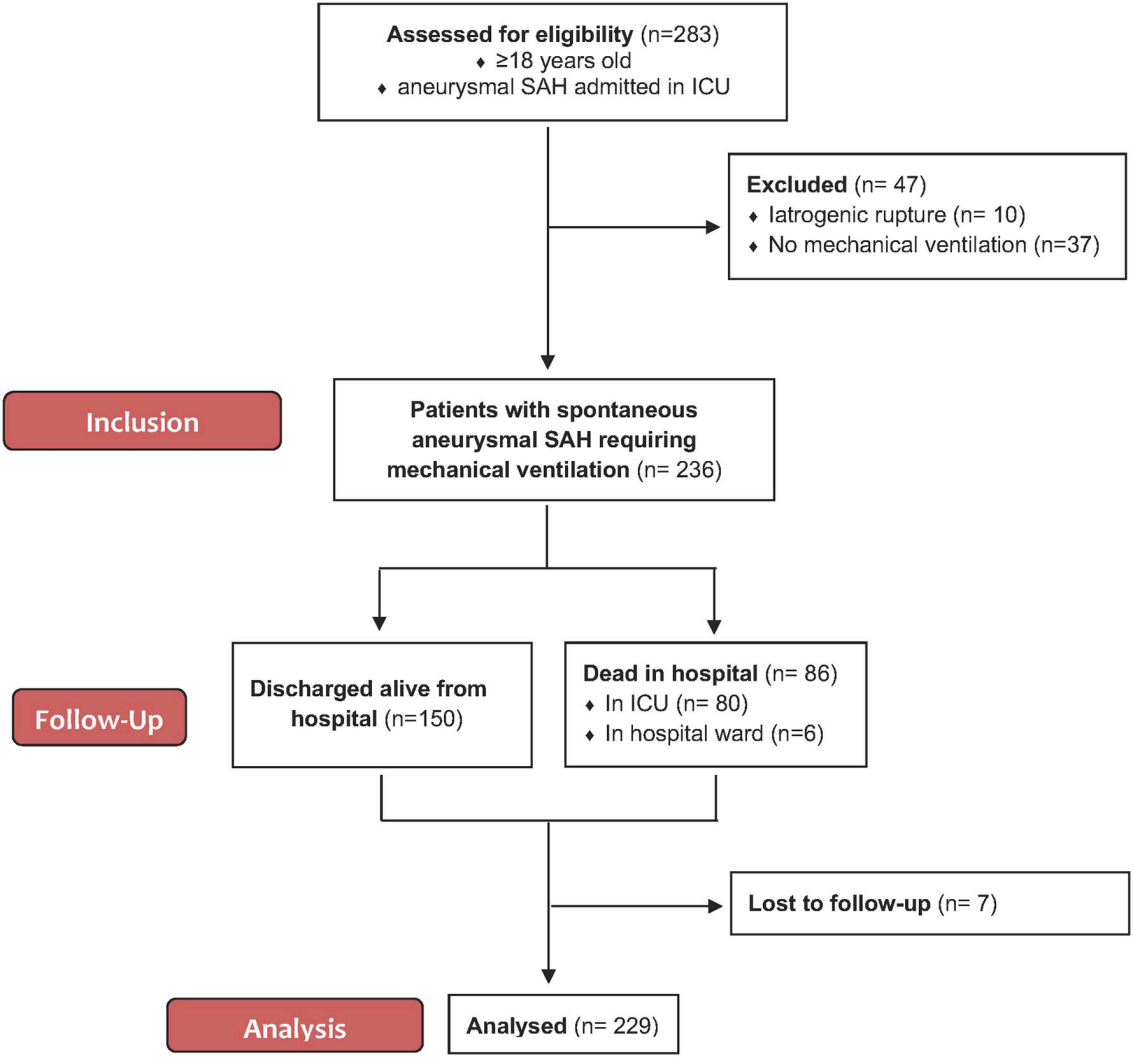
Flow chart of the study design. SAH subarachnoid hemorrhage, ICU Intensive Care Unit.

The flow chart of the study design is shown in Fig 1. Two hundred and thirty-six patients were admitted to the ICU for spontaneous aSAH requiring mechanical ventilation. Seven patients were lost to follow-up. The demographic and clinical characteristics of the 229 analysed patients are summarized in Table 1. Subjects mean age was 55±13 years, female represented 63% of patients. Poor grade aSAH (WFNS IV and V) constituted ninety-one percent of cases. All patients were mechanically ventilated from the time of ICU admission. Two hundred and twenty-two (97%) patients were ventilated for at least 48 hours. Mean mechanical ventilation and ICU stay durations were 22±17 and 33±23 days in patients discharged alive form the ICU, and were 11±14 and 12±15 days in patients who passed.

**Table 1:** Description of patients with critical subarachnoid hemorrhage requiring mechanical ventilation. Variables comparison between the good- and poor-outcome groups.

Variable	Overall (N = 229)	Good-Outcome (N = 73)	Poor-Outcome (N = 176)	Difference (95% CI)
**Age**, years	55 ± 13	51.70 ±13.53	56.48 ± 12.57	4.78 (1.15; 8.41)
**Male sex, n (%)**	84 (37%)	22 (30.14%)	62 (39.74%)	9.61 (-3.42; 22.64)
**Tobacco use, n (%**	75 (33%)	30 (41.10%)	45 (28.85%)	-12.25 (-25.59; 1.09)
**GCS on admission**				
≤5	88 (42%)	21 (32.81%)	67 (46.53%)	13.72 (-0.38; 27.81)
6–8	69 (33%)	19 (29.69%)	50 (34.72%)	5.03 (-8.59; 18.66)
≥9	51 (25%)	24 (37.50%)	27 (18.75%)	-18.75 (-32.22; -5.28)
**SAPSII on admission**	42.3 ± 12	37.80 ± 10.38	44.46 ± 12.45	6.67 (3.39; 9.94)
**WFNS score, n (%)**				
III	21 (9%)	11 (15.07%)	10 (6.41%)	-8.66 (-17.72; 0.40)
IV	94 (41%)	34 (46.58%)	60 (38.46%)	-8.11 (-21.87; 5.64)
V	114 (50%)	28 (38.36%)	86 (55.13%)	16.77 (3.16; 30.39)
**Intracerebral hemorrhage, n (%)**	105 (46%)	28 (38.36%)	77 (49.36%)	11.00 (-2.63; 24.64)
**Fisher Score, n (%)**				
I	1	1 (1.37%)	0	-1.37 (-4.04; 1.30)
II	5 (2%)	3 (4.11%)	2 (1.28%)	-2.83 (-7.71; 2.06)
III	43 (19%)	16 (21.92%)	27 (17.31%)	-4.61 (-15.80; 6.58)
IV	180 (79%)	53 (72.60%)	127 (81.41%)	8.81 (-3.11; 20.72)
**Hydrocephalus requiring EVD, n (%)**	156 (68%)	46 (63.01%)	110 (70.51%)	7.50 (-5.69; 20.68)
**Aneurysm-securing procedure, n (%)**				
Coiling	145 (63%)	54 (73.97%)	91 (58.33%)	-15.64 (-28.33; -2.94)
Clipping	60 (26%)	18 (24.66%)	42 (26.92%)	-15.64 (-28.33; -2.94)
Both	4 (2%)	1 (1.37%)	3 (1.92%)	-15.64 (-28.33; -2.94)
No treatment (Failure/Withdrawal of care)	20 (9%)	0	20 (12.82%)	12.82 (7.57; 18.07)
**Aneurysm-securing delay, n (%)**				
<12 hours	129 (56%)	43 (58.90%)	86 (55.13%)	-3.78 (-17.50; 9.95)
12 h–24 hours	65 (28%)	26 (35.62%)	39 (25.00%)	-10.62 (-23.53; 2.30)
24–48 hours	9 (4%)	2 (2.74%)	7 (4.49%)	1.75 (-3.21; 6.70)
>48 hours	6 (3%)	2 (2.74%)	4 (2.56%)	-0.18 (-4.67; 4.32)
Failure	7 (3%)	0	7 (4.49%)	-0.18 (-4.67; 4.32)
Withdrawal of care	13 (6%)	0	13 (8.33%)	8.33 (4.00; 12.67)
**Early brain injuries, n (%)**	163 (71%)	36 (49.32%)	127 (81.94%)	32.62 (19.65; 45.59)
**Rebleeding, n (%)**	33 (14%)	2 (2.74%)	31 (19.87%)	17.13 (9.84; 24.43)
**Angiographic vasospasm, n (%)**	80 (35%)	22 (30.14%)	58 (37.18%)	7.04 (-5.93; 20.02)
**Delayed cerebral ischemia, n (%)**	63 (27%)	14 (19.18%)	49 (31.41%)	12.23 (0.63; 23.83)
**Delay of mRS assessment**, months	24 ± 25	41.20 ± 22.25	16.34 ± 23.04	-24.86 (-31.60;-18.12)

Description of the 229 patients with critical aneurysmal subarachnoid hemorrhage. Comparison of variables between the good- and poor-outcome groups (multivariable analysis). Data are expressed as the means ± SD. aSAH indicates Aneurysmal Subarachnoid Hemorrhage; GCS, Glasgow Coma Scale; SAPSII, Simplified Acute Physiology Score II; WFNS, World Federation of Neurological Surgeons; EVD, External Ventricular Drain; mRS, modified Rankin Score; ICU, Intensive Care Unit.

### Neurological events

Among the 229 patients, 163 (71%) presented EBI. Thirty-three patients (14%) rebled, eleven before, 7 during and 15 (8%) after the aneurysm securing procedure. Angiographic vasospasm and DCI were diagnosed in 80 (35%) and 64 (27%) patients respectively.

Among the 115 patients who appeared to be in poor grade on admission (WFNS V patients), 69 (60%) of them were treated with EVD. None of them of showed sufficient improvement for early mechanical ventilation weaning. One third of them (38/115) had good outcome (mRS<3).

### Rescue therapy for delayed cerebral ischemia

Intra-arterial nimodipine and intra-arterial milrinone were administered to 58/80 (72.5%) patients presenting angiographic vasospasm, in a mean total sessions of 2.86 ±1.6. PTA was performed in 22/80 (27.5%) patients. Twenty-nine (36.25%) patients received intravenous milrinone at low dose.

### Survival

**Fig 2:**
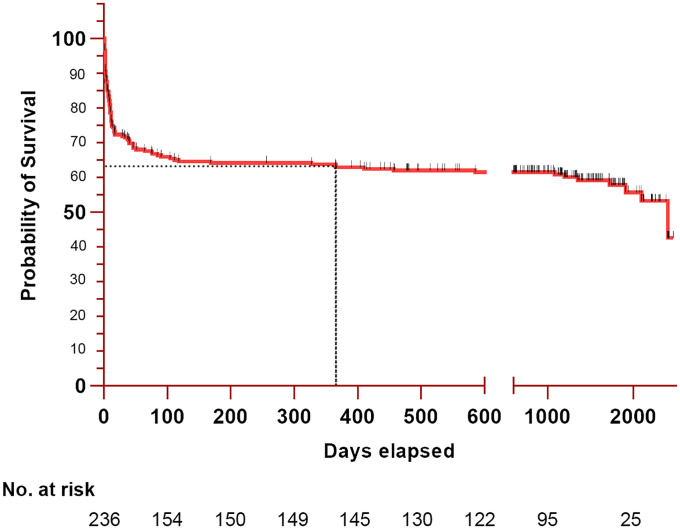
Kaplan-Meier estimates of survival after intensive care unit admission of patients with critical aneurysmal subarachnoid hemorrhage requiring mechanical ventilation. The estimated survival of 236 patients after ICU admission for critical aSAH. The median survival length was 20 months. The estimated one-year survival (indicated by the dotted line) was 63.08%.

Eighty patients (33.9%) died during the ICU stay. Six patients (2.5%) deceased at the hospital after being discharged from the ICU, and 12 (5.1%) after discharge from the hospital. Among the 80 deaths occuring in the ICU, 38 (47.5%) happened within the first 5 days, 58 (72.5%) resulted from brain death or refractory intracranial hypertension, 19 (23.75%) followed withdrawal of care and 3 (3.75%) were attributable to other causes (e.g., cardiorespiratory failure). The median survival length was 20.6 [0.4–48.7] months, and the estimated one-year survival rate was 63.1±3.1%. The Kaplan-Meier survival curve is shown in Fig 2.

### Long-term neurological outcome

**Fig 3:**
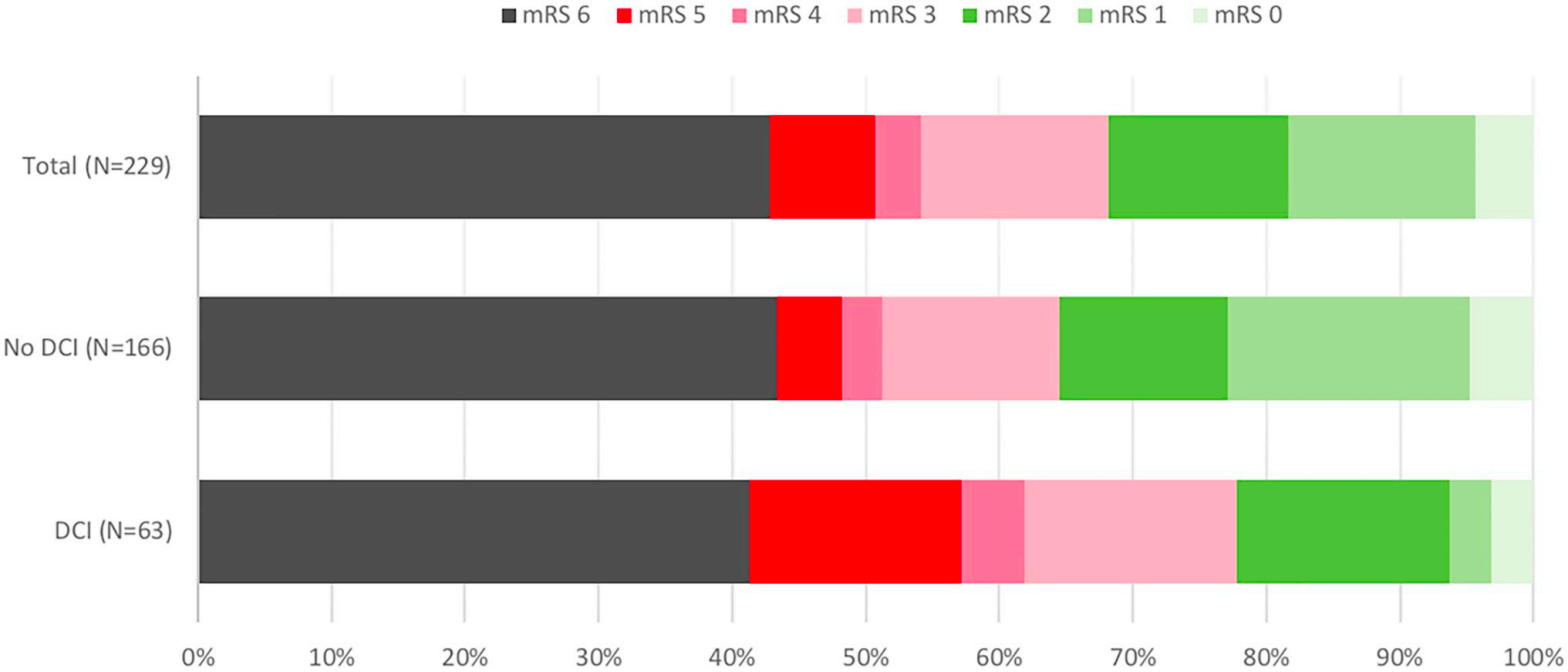
Long-term outcome for the total population, and for subgroups diagnosed or not with delayed cerebral ischemia. Modified Rankin Score (mRS) was assessed with a standardized phone survey at least one year after initial bleeding. The degree of disability or dependence for daily activities was measured on a scale ranging from 0 to 6: 0—No symptom; 1—No significant disability despite some symptoms; 2—Slight disability (unable to perform all of former activities but no dependence); 3—Moderate disability (help required for ordinary tasks but walking conserved without the need for assistance); 4—Moderately severe disability (inability to walk without assistance); 5—Severe disability (constantly bedridden); 6—Death. Among survivors, the proportions of patients with moderate to severe disabilities (mRS 3-5—shown in red colors) and with mRS 0-2 were compared to each other, in the “DCI” and “No DCI” subgroups using a chi-square test.

Seven patients (3%) were lost to follow-up (Fig 1). Long-term neurological outcome of the 229 remaining patients is displayed in Fig 3. Seventy-three (32%) patients had good outcome (mRS<3), in detail: 10 (4%) had no symptom (mRS = 0), 32 (14%) showed symptoms but no disability (mRS = 1), 31 (14%) endured slight disability (mRS = 2), 32 (14%) suffered moderate disability (mRS = 3), 8 (3%) patients were affected by moderately severe disability (mRS = 4), 18(8%) were afflicted by severe disability (mRS = 5) and 98 (43%) patients had deceased. Eighteen patients (8%) underwent gastrostomy and tracheostomy insertion, six patients underwent tracheostomy alone (3%) and 4 patients gastrostomy alone (2%). Among the 131 long-term survivors, the proportion of patients with moderate to severe disability (mRS 3―5) was significantly higher in patients diagnosed with DCI than in those who were not (Fig 3): 23/37 (62.16%) versus 35/94 (37.23%), p = 0.0183). The mean delay between SAH diagnosis and mRS assessment (phone call or date of death) was 27±26 months, and was shorter in the poor-outcome group (16.34±23.04 versus 44.05±20.47 months, p<0.001).

### Factors associated with poor outcome

Demographic and clinical variables in the good- and poor-outcome groups were compared to each other. The univariable analysis results are shown in Table 1. Among variables defined based on evaluation at admission, mean age, SAPSII and WFNS scores were significantly higher in the poor-outcome group, and GCS was significantly higher in the good-outcome group. There were significantly more cases of EBI, rebleeding and clipping therapeutic in the poor-outcome group. The time to mRS assessment was shorter in the poor-outcome group. The occurrences of vasospasm or DCI were not statistically associated with poor-outcome in the univariable analysis.

In the multivariable logistic regression model, of which the results are shown in Table 2, factors independently associated with poor-outcome were time to mRS assessment (OR per month, 0.96; 95% CI, 0.95-0.98; p<.0001), age (OR per 10 years, 1.57; 95% CI, 1.12-2.19; p = 0.008), WFNS V versus WFNS III (OR, 5.71; 95% CI 1.51-21.61; p = 0.004), subarachnoid rebleeding (OR, 6.47; 95% CI 1.16-36.06; p = 0.033), EBI (OR, 4.52; 95% CI 1.81-11.29; p = 0.001) and DCI (OR, 4.73; 95% CI, 1.66-13.49; p = 0.004). The goodness of fit was evaluated with the Hosmer-Lemeshow test (p = 0;5776).

**Table 2:** Results of a multivariable logistic regression model for variables associated with poor long-term outcome (mRS 3-6).

Variable	Odds Ratio (95% CI)	p-value
**Delay of mRS assessment** (per month)	0.96 (0.95; 0.98)	<0.0001
**Age** (per 10 years)	1.57 (1.12; 2.19)	0.008
**WFNS V versus WFNS III**	5.71 (1.51; 21.61)	0.004
**WFNS IV versus WFNS III**	2.15 (0.59; 7.87)	
**Rebleeding**	6.47 (1.16; 36.06)	0.033
**Early brain injuries**	4.52 (1.81; 11.29)	0.001
**Delayed cerebral ischemia**	4.73 (1.66; 13.49)	0.004

## Discussion

In the large MASH cohort, we estimated the long term outcome in aSAH patients using the mRS score. In line with data available in the literature, the one-year survival rate was 63.1±3.1%, and poor outcome affected 68% of patients. The risk factors for poor outcome were also extracted, using a multivariable logistic regression model. Among these, age and WFNS score are unmodifiable in patients, rebleeding and EBI are managed through early aneurysm exclusion and non-specific neurocritical care. Time-to-assessment and DCI, identified as protective and risk factors respectively, are discussed in the following paragraphs.

This study presents some limitations, particularly hindering the interpretation of the time-to assessment factor. Indeed, it appears as an independent protective factor, though this effect might arise from bias due to early evaluation of the 36.4% of patients with mRS6, i.e. who passed, during hospital stay. Alternatively, it could result from an improvement of patients status with time, as suggested in a recent study ([11]). This ambiguity stems from the main limitation of this study, that is the absence of outcome evaluation as a function of treatment and time. Indeed, clinicians practice may have changed during the study period, and time-to-assessment was not set as a function of aSAH onset, so that the study population lacks homogeneity. The retrospective nature of the study hindered the gathering of such information. Nonetheless, clinical outcome in ICU patients have be assessed, with a very low lost to follow-up rate <3%.

Finally, DCI was identified as an independent risk factor for poor outcome, that lacks specific treatment evaluation. Even though large randomized controlled trials would be ideal for fulfilling that latter goal, we are still missing clear pathophysiological target. In fact, angiographic vasospasm was not statistically associated with poor outcome in this study. This result is in accordance with other data from the literature, showing 1) a mismatch between angiographic vasoconstriction and clinical DCI, diagnosed in 70 and 30% of aSAH patients respectively, 2) delayed cerebral infarction independently from angiographic vasoconstriction, 3) poor temporal relationship between ultrasonographic vasospasm and DCI, 4) poor correlation between successful treatment of angiographic vasospasm and outcome improvement [2, 16, 18]. These observations have led to other conceptions of DCI, focused on mid-term EBI consequences. Briefly, early cell death, increased ICP, acute hydrocephalus, brain oedema, acute vasoconstriction, damage to the blood-brain barrier, impaired autoregulation, microthrombosis, inflammation, and cortical spreading ischaemia have all been proposed to play a role in DCI pathogenesis [18]. Of particular interest are the strong arguments for microvasculature involvement [16, 32, 33], showing early and long-lasting vasoconstriction and thrombosis in animal models [34]. In our view, this lead should be followed through, calling for both experimental and clinical studies in order to unravel the mechanisms of this dreadful complication.

## Conclusion

This study describes long-term outcome in a large cohort of critically ill patients afflicted with aSAH, and provides accurate data on factors associated with disability and mortality. Though this latter remains high, one-third of patients have a good functional outcome. As it appears as a singular potentially treatable factor, DCI should certainly retain attention beyond angiographic vasospasm in future studies.

## References

[1] Suarez JI, Tarr RW, Selman WR. Aneurysmal Subarachnoid Hemorrhage. New England Journal of Medicine. 2006;354(4):387–396. 10.1056/NEJMra052732 16436770

[2] Macdonald RL. Delayed neurological deterioration after subarachnoid haemorrhage. Nature Reviews Neurology. 2014;10(1):44–58. 10.1038/nrneurol.2013.246 24323051

[3] Hop JW, Rinkel GJE, Algra A, Gijn Jv. Case-Fatality Rates and Functional Outcome After Subarachnoid Hemorrhage: A Systematic Review. Stroke. 1997;28(3):660–664. 10.1161/01.STR.28.3.660 9056628

[4] Nieuwkamp DJ, Setz LE, Algra A, Linn FHH, de Rooij NK, Rinkel GJE. Changes in case fatality of aneurysmal subarachnoid haemorrhage over time, according to age, sex, and region: a meta-analysis. Lancet Neurol. 2009;8(7):635–642. 10.1016/S1474-4422(09)70126-7 19501022

[5] Udy AA, Vladic C, Saxby ER, Cohen J, Delaney A, Flower O, et al. Subarachnoid Hemorrhage Patients Admitted to Intensive Care in Australia and New Zealand: A Multicenter Cohort Analysis of In-Hospital Mortality Over 15 Years. Crit Care Med. 2017;45(2):e138–e145. 10.1097/CCM.0000000000002059 27749342

[6] Molyneux AJ, Kerr RS, Birks J, Ramzi N, Yarnold J, Sneade M, et al. Risk of recurrent subarachnoid haemorrhage, death, or dependence and standardised mortality ratios after clipping or coiling of an intracranial aneurysm in the International Subarachnoid Aneurysm Trial (ISAT): long-term follow-up. The Lancet Neurology. 2009;8(5):427–433. 10.1016/S1474-4422(09)70080-8 19329361 PMC2669592

[7] Al-Khindi T, Macdonald RL, Schweizer TA. Cognitive and Functional Outcome After Aneurysmal Subarachnoid Hemorrhage. Stroke. 2010;41(8). 10.1161/STROKEAHA.110.581975 20595669

[8] Cinotti R, Putegnat JB, Lakhal K, Desal H, Chenet A, Buffenoir K, et al. Evolution of neurological recovery during the first year after subarachnoid haemorrhage in a French university centre. Anaesth Crit Care Pain Med. 2019;38(3):251–257. 10.1016/j.accpm.2018.10.002 31079704

[9] de Oliveira Manoel AL, Mansur A, Silva GS, Germans MR, Jaja BNR, Kouzmina E, et al. Functional Outcome After Poor-Grade Subarachnoid Hemorrhage: A Single-Center Study and Systematic Literature Review. Neurocritical Care. 2016;25(3):338–350. 10.1007/s12028-016-0305-3 27651379

[10] Inamasu J, Nakae S, Ohmi T, Kogame H, Kawazoe Y, Kumai T, et al. The outcomes of early aneurysm repair in World Federation of Neurosurgical Societies grade V subarachnoid haemorrhage patients with emphasis on those presenting with a Glasgow Coma Scale score of 3. Journal of Clinical Neuroscience. 2016;33:142–147. 10.1016/j.jocn.2016.03.035 27450281

[11] Schwartz C, Pfefferkorn T, Ebrahimi C, Ottomeyer C, Fesl G, Bender A, et al. Long-term Neurological Outcome and Quality of Life after World Federation of Neurosurgical Societies Grades IV and V Aneurysmal Subarachnoid Hemorrhage in an Interdisciplinary Treatment Concept. Neurosurgery. 2017;80(6):967–974. 10.1093/neuros/nyw138 28327912

[12] Galea JP, Dulhanty L, Patel HC. Predictors of Outcome in Aneurysmal Subarachnoid Hemorrhage Patients: Observations From a Multicenter Data Set. Stroke. 2017;48(11):2958–2963. 10.1161/STROKEAHA.117.017777 28974630

[13] Schmidt JM, Rincon F, Fernandez A, Resor C, Kowalski RG, Claassen J, et al. Cerebral infarction associated with acute subarachnoid hemorrhage. Neurocrit Care. 2007;7(1):10–17. 10.1007/s12028-007-0003-2 17657652

[14] Gonçalves B, Turon R, Mendes A, Melo N, Lacerda P, Brasil P, et al. Effect of Early Brain Infarction After Subarachnoid Hemorrhage: A Systematic Review and Meta-Analysis. World Neurosurgery. 2018;115:e292–e298. 10.1016/j.wneu.2018.04.037 29660554

[15] Cahill WJ, Calvert JH, Zhang JH. Mechanisms of Early Brain Injury after Subarachnoid Hemorrhage. Journal of Cerebral Blood Flow & Metabolism. 2006; p. 13. 16482081 10.1038/sj.jcbfm.9600283

[16] de Oliveira Manoel AL, Goffi A, Marotta TR, Schweizer TA, Abrahamson S, Macdonald RL. The critical care management of poor-grade subarachnoid haemorrhage. Critical Care. 2016;20(1). 10.1186/s13054-016-1193-9 26801901 PMC4724088

[17] Vergouwen MDI, Vermeulen M, Gijn Jv, Rinkel GJE, Wijdicks EF, Muizelaar JP, et al. Definition of Delayed Cerebral Ischemia After Aneurysmal Subarachnoid Hemorrhage as an Outcome Event in Clinical Trials and Observational Studies Proposal of a Multidisciplinary Research Group. Stroke. 2010;41(10):2391–2395. 10.1161/STROKEAHA.110.589275 20798370

[18] Rowland MJ, Hadjipavlou G, Kelly M, Westbrook J, Pattinson KTS. Delayed cerebral ischaemia after subarachnoid haemorrhage: looking beyond vasospasm. British Journal of Anaesthesia. 2012;109(3):315–329. 10.1093/bja/aes264 22879655

[19] Ayling OG, Ibrahim GM, Alotaibi NM, Gooderham PA, Macdonald RL. Dissociation of Early and Delayed Cerebral Infarction After Aneurysmal Subarachnoid Hemorrhage. Stroke. 2016;47(12):2945–2951. 10.1161/STROKEAHA.116.014794 27827324

[20] Frontera JA, Fernandez A, Schmidt JM, Claassen J, Wartenberg KE, Badjatia N, et al. Defining vasospasm after subarachnoid hemorrhage: what is the most clinically relevant definition? Stroke. 2009;40(6):1963–1968. 10.1161/STROKEAHA.108.544700 19359629

[21] Connolly ES, Rabinstein AA, Carhuapoma JR, Derdeyn CP, Dion J, Higashida RT, et al. Guidelines for the Management of Aneurysmal Subarachnoid Hemorrhage: A Guideline for Healthcare Professionals From the American Heart Association/American Stroke Association. Stroke. 2012;43(6):1711–1737. 10.1161/STR.0b013e3182587839 22556195

[22] Lindegaard KF, Nornes H, Bakke SJ, Sorteberg W, Nakstad P. Cerebral vasospasm after subarachnoid haemorrhage investigated by means of transcranial Doppler ultrasound. Acta Neurochir Suppl (Wien). 1988;42:81–84. 3055838 10.1007/978-3-7091-8975-7_16

[23] D’Andrea A, Conte M, Scarafile R, Riegler L, Cocchia R, Pezzullo E, et al. Transcranial Doppler Ultrasound: Physical Principles and Principal Applications in Neurocritical Care Unit. Journal of Cardiovascular Echography. 2016;26(2):28–41. 10.4103/2211-4122.183746 28465958 PMC5224659

[24] Fraticelli AT, Cholley BP, Losser MR, Saint Maurice JP, Payen D. Milrinone for the treatment of cerebral vasospasm after aneurysmal subarachnoid hemorrhage. Stroke; a Journal of Cerebral Circulation. 2008;39(3):893–898. 10.1161/STROKEAHA.107.492447 18239182

[25] Romero CM, Morales D, Reccius A, Mena F, Prieto J, Bustos P, et al. Milrinone as a rescue therapy for symptomatic refractory cerebral vasospasm in aneurysmal subarachnoid hemorrhage. Neurocrit Care. 2009;11(2):165–171. 10.1007/s12028-008-9048-0 18202923

[26] Bashir A, Andresen M, Bartek JJ, Cortsen M, Eskesen V, Wagner A. Intra-arterial nimodipine for cerebral vasospasm after subarachnoid haemorrhage: Influence on clinical course and predictors of clinical outcome. Neuroradiol J. 2016;29(1):72–81. 10.1177/1971400915626429 26825134 PMC4978346

[27] Hoh BL, Ogilvy CS. Endovascular Treatment of Cerebral Vasospasm: Transluminal Balloon Angioplasty, Intra-Arterial Papaverine, and Intra-Arterial Nicardipine. Neurosurgery Clinics of North America. 2005;16(3):501–516. 10.1016/j.nec.2005.04.004 15990041

[28] Lannes M, Teitelbaum J, del Pilar Cortés M, Cardoso M, Angle M. Milrinone and Homeostasis to Treat Cerebral Vasospasm Associated with Subarachnoid Hemorrhage: The Montreal Neurological Hospital Protocol. Neurocritical Care. 2012;16(3):354–362. 10.1007/s12028-012-9701-5 22528278

[29] Rankin J. Cerebral vascular accidents in patients over the age of 60. II. Prognosis. Scott Med J. 1957;2(5):200–215. 10.1177/003693305700200504 13432835

[30] Bruno A, Akinwuntan AE, Lin C, Close B, Davis K, Baute V, et al. Simplified modified rankin scale questionnaire: reproducibility over the telephone and validation with quality of life. Stroke. 2011;42(8):2276–2279. 10.1161/STROKEAHA.111.613273 21680905

[31] Toulouse E, Masseguin C, Lafont B, McGurk G, Harbonn A, Roberts JA, et al. French legal approach to clinical research. Anaesthesia Critical Care & Pain Medicine. 2018;37(6):607–614. 10.1016/j.accpm.2018.10.013 30580775

[32] Østergaard L, Aamand R, Karabegovic S, Tietze A, Blicher JU, Mikkelsen IK, et al. The Role of the Microcirculation in Delayed Cerebral Ischemia and Chronic Degenerative Changes after Subarachnoid Hemorrhage. Journal of Cerebral Blood Flow & Metabolism. 2013;33(12):1825–1837. 10.1038/jcbfm.2013.173 24064495 PMC3851911

[33] Terpolilli NA, Brem C, Bühler D, Plesnila N. Are We Barking Up the Wrong Vessels?: Cerebral Microcirculation After Subarachnoid Hemorrhage. Stroke. 2015;46(10):3014–3019. 10.1161/STROKEAHA.115.006353 26152299

[34] Friedrich B, Müller F, Feiler S, Schöller K, Plesnila N. Experimental Subarachnoid Hemorrhage Causes Early and Long-Lasting Microarterial Constriction and Microthrombosis: An *in-vivo* Microscopy Study. Journal of Cerebral Blood Flow & Metabolism. 2012;32(3):447–455. 10.1038/jcbfm.2011.154 22146194 PMC3293113

